# The effect of zinc therapy on electrolyte balance in children with acute diarrhea

**DOI:** 10.1186/1471-2334-13-S1-P61

**Published:** 2013-12-16

**Authors:** Nicoleta Negruț

**Affiliations:** 1 Third Department of Pediatrics, Iuliu Hațieganu University of Medicine and Pharmacy, Cluj-Napoca, Romania; 2 Department of Neuroscience and Recovery, Faculty of Medicine and Pharmacy, University of Oradea, Romania

## Background

Electrolyte disturbances in acute diarrhea may occur with insufficient intake or excessive loss of ions. Treatment with zinc salt was recommended by WHO and UNICEF since 2008 for children under 5 years old.

## Methods

During 2009-2011, children with acute diarrhea were enrolled in a randomized, open comparative study. Zinc salt was given to the patients from the study group according to WHO recommendations. The outcomes were the serum sodium, potassium and chloride levels in days 1, 2 and 3 of zinc therapy. Data were processed using EPIINFO version 6.0.

## Results

In the above-mentioned time span 116 children, 0-3 years old, were enrolled. A total of 103 children recruited were available for analysis. In the study group (n=53) compared with the control group (n=50), there was no significant difference in serum sodium, potassium and chloride level (p > 0.05), on days 1, 2, and 3. Similarly, there was no significant difference between the concentrations of electrolytes in the two groups followed daily.

## Conclusion

Zinc therapy in acute diarrhea does not seem to influence serum electrolytes in children under 3 years old.

